# The Effect of *Taraxacum officinale* Hydroalcoholic Extract on Blood Cells in Mice

**DOI:** 10.1155/2012/653412

**Published:** 2012-07-12

**Authors:** Mehrdad Modaresi, Narges Resalatpour

**Affiliations:** ^1^Department of Agriculture, Islamic Azad University, Khorasgan Branch, Isfahan, Iran; ^2^Isfahan Center, Payame Noor University, Isfahan, Iran

## Abstract

*Objectives*. Dandelion (*Taraxacum officinale*) is a herbaceous perennial plant of the family Asteraceae and has medicinal and culinary uses. Dandelion has been used as a remedy for anemia, purifing the blood, and providing immune modulation. Therefore, the aim of this study was to investigate the effect of hydro alcoholic extract on blood cells in mice. *Methods*. Five groups each including ten adult female (Balb/C) mice weighing 30 ± 5 g were chosen. Normal saline was administered as placebo for group, and dandelion hydro alcoholic extract in doses of 50,100, and 200 mg/kg was injected intraperitoneally for 20 days to test groups and the last group was control group.WBC, RBC, HB, HCT, platelet, and other cells were measured with automated cell counter. *Main Results*. The number of RBC and the rate of HB in three doses of 100 and 200 mg/kg significantly increased (*P* < 0.05). As compared with control group, the number of WBC in three doses of 50, 100, and 200 mg/kg increased, but it was significantly in 200 mg/kg dandelion treated group as compared with control group(*P* < 0.05). The rate of platelet in three doses of 50, 100 and 200 mg/kg significantly decreased as compared with control group (*P* < 0.01). 3 doses of dandelion increased lymphocyte numbers significantly compared with controls. *Conclusion*. The study indicates efficacy of dandelion extract on RBC and HB in doses of 50, 100, and 200 mg/kg and in 200 mg/kg on WBC to achieve normal body balance.

## 1. Introduction

History of using herbs to treat diseases and health gain has been common in human societies. Many researchers in last years had proved that herbs such as dandelion have considerable effects on treatment of disease. *Taraxacum officinale* is from the family Asteraceae [1].This hardly perennial herb usually has deeply toothed. Hairless leaves, 5–30 cm long and 1–10 cm wide. It grows 3–35 cm in height, forming a rosette of leaves at ground level. It has single, golden yellow flowers on straight. Leafless hollow stems, which emerge from the centre of the rosette. Each flower consists of a collection of florets. Flowers are produced from early spring until late autumn. When the florets mature, they produce downy seeds, which are easily dispersed by the wind [2] Dandelion plants have tap roots, tapering from 2 to 3 cm wide and at least 15 cm in length. Roots are fleshy and brittle, and are a dark brown color on the outside and white on the inside. Dandelion, *Taraxacum officinale*, was native to Europe but now can be found throughout the northern temperate zones [2] Dandelion is a rich source of vitamins and minerals and is particularly high in vitamins A and C and iron, carrying more iron and calcium than spinach [2] The folk medicines of China, India, and Russia have recognized dandelion's effect as a liver tonic. Traditional Chinese medicine combines dandelion with other herbs to treat hepatitis [3], they used it to enhance the immune response to upper respiratory tract infections, bronchitis and pneumonia, and as a topical compress to treat mastitis [4]. Also it is used in the treatment of anemia and inflammation [5]. It used in treatment of jaundice, toxity, purifying the blood, fever, eye problems, gastrointestinal problems osteoarthritis, eczema, and cancer of uterus and breast in women [3].

## 2. Materials and Methods

Five groups each including ten adult female (Balb/C) mice weighing 30 ± 5 g were chosen. Normal saline was administered as placebo for group and dandelion hydro alcoholic extract in doses of 50, 100, and 200 mg/kg was injected intraperitoneally for 20 days to test groups and the last group was control group. WBC, RBC, HB, HCT, platelet, and other cells were measured with automated cell counter. For the statically calculation, we used ANOVAs and Duncan tests and the amount of *P* < 0.05 were considered significant.

To prepare the extract of dandelion, they were grinded completely and 30 g of obtained powder was poured in a sterilized Erlenmeyer flask, 40 cc of physiological serum was added to it, and was located in a cool place. After 24 hours, Erlenmeyer flask contents were mixed completely using a shaker for five minutes. Then, after filtering the solution by filteration paper and calculating extract residual in solution, concentration of extract in base solution was determined, and doses were prepared.

## 3. Results and Discussion

**Figure 1:**
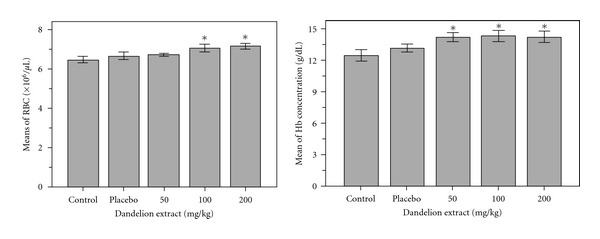
Comparison of average number of red blood cells and mean hemoglobin concentrations between experimental groups, control and placebo.

Comparison of red blood cells between control and experimental groups showed significant increase of red blood cells in experimental group 2 (treated with a dose 100 mg/kg) and 3 (treated with a 200 mg/kg) than the control group (*P* < 0.05).  Figure 1 shows the results of this study.

**Figure 2:**
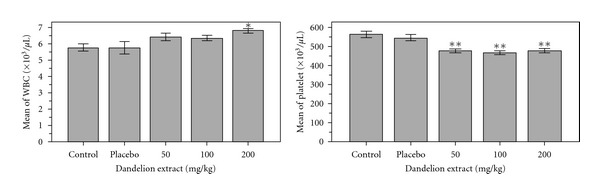
Comparison of mean platelet and mean number of white blood cells between experimental groups, control and placebo.

Experimental study of hemoglobin in the control groups with experimental groups showed significant increases in mean hemoglobin in group 1 (treated with a dos 50 mg/kg), 2 (treated with a dose 100 mg/kg) and 3 (treated with a dose 200 mg/kg) compared to control (*P* < 0.05).  Figure 1 shows the result of this study. The average hematocrit in the blood of control groups compared with experimental groups showed a hematocrit level in group 1 (treated with a does 50 mg/kg), group 2 (treated with a dose 100 mg/kg) and 3 (treated with a dose 200 mg/kg) are lower than the control group but these differences aren't be significant. Conducted study showed that the average percentage of MCV in group 1 (treated with a dose 50 mg/kg), 2 (treated with a dose 100 mg/kg) and 3 (treated with a dose 200 mg/kg) increased than the control group, but these differences arenot significant. The average of MCH between control and experimental groups showed that the amount of MCH in group 1 (treated with a dose 50 mg/kg), group 2 (treated with a dose100 mg/kg) increased than the control group and in group 3 (treated with a dose 200 mg/kg) decreased but these differences arenot significant. The average percentage of MCHC in the blood of mice's experimental group 2 (treated with a dose 100 mg/kg) and 3 (treated with a dose 200 mg/kg) increased slightly compared to the control group and group 1 (treated with a dose 50 mg/kg) showed a minor decrease. But none of these differences aren't significant. Study shows significant decrease in blood platelet levels in the experimental groups 1 (treated with a dose 50 mg/kg), 2 (treated with a dose 100 mg/kg) and 3 (treated with a dose 200 mg/kg) compared to control group (*P* < 0.01).  Figure 2 shows the results of this study.

**Figure 3:**
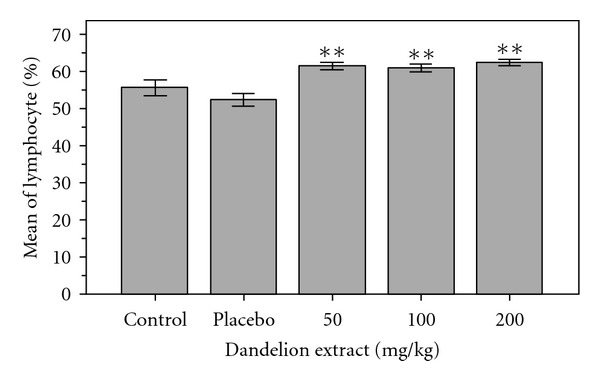
Comparison of mean lymphocytes between the experimental groups, control and placebo

Comparison of average number of white blood cells in the control group compared with experimental groups 1 (treated with a dose 50 mg/kg), 2 (treated with a dose 100 mg/kg), and 3 (treated with a dose 200 mg/kg) indicated increasing compared with control group, but this difference only in group 3 (treated with a dose 200 mg/kg) is significant (*P* < 0.05).  Figure 2 shows the results of this study. Average percentage of control blood neutrophils with the experimental groups showed that rate of neutrophils experimental groups 1 (treated with a dose 50 mg/kg), 2 (treated with a dose 100 mg/kg), and 3 (treated with a dose 200 mg/kg) have shown a slight reduction compared to control group, But neither in the experimental group nor the control group, a difference is not significant. Average percentage of lymphocytes between the control group compared with the experimental groups 1 (treated with a dose 50 mg/kg), 2 (treated with a dose 100 mg/kg) and 3 (treated with a dose 200 mg/kg) indicated a significant increase (*P* < 0.01).  Figure 3 shows the results of this study.

As in Figure 1 was shown, the amount of red blood cells in experimental group 2 (treated with a dose 100 mg/kg) and 3 (treated with a dose 200 mg/kg)and the amount of hemoglobin in groups 1 (treated with a dose 50 mg/kg), 2 and 3 than the control group had increased significantly. This could be due to the positive effect of dandelion on the liver [6] and increased secretion of erythropoietin (a substance that regulates the amount of red blood cell) per. As research, phenolic compounds in dandelion such as: chicoric acidact as an antioxidant by preventing the oxidation of collagen and cells and inhibits the penetration of viruses in cells, and chlorogenic acid is cholagogue: its regular ingestion helps the flow of bile and thus reduces the adverse effects of bile stagnation [7]. These compound, are the most important compound that explain the positive effect of dandelion on liver. The prebiotics are nondigestible carbohydrates, many of these carbohydrates are short chains of monosaccharide called oligosaccharides. Some oligosaccharides are thought to enhance the growth of beneficial organisms in the gut, and others are thought to function as competitive attachment sites for pathogenic bacteria. Therefore, dandelion could be used as a main source of inulin, and it is used as a prebiotic. Also antioxidant effect of Dandelion belongs to phenolic, flavonoids and coumaric acid compounds [8]. These compounds can protect membranes like RBC membrane from injury of free radicals and prevent from their hurt and increase number in blood.  Figure 2 showed that the amount of platelet in experimental groups 1 (treated with a dose 50 mg/kg), 2 (treated with a dose 100 mg/kg), and 3 (treated with a dose 200 mg/kg) than the control had a significant reduction. According to studies unsaturated fatty acids like linolenic acid present in *Taraxacum officinale* increase linolenic in blood platelet and reduce not only thromboxane synthesis but also aggregation, in this way reducing the thrombosis possibility [9]. Fatty acids modulate immune responses through one or more of three major molecular mechanisms: (1) altered membrane composition and function, (2) modified eicosanoid production, and (3) changed cytokine biosynthesis. Also coumarins in *Taraxacum officinale* act as anticoagulation and prevent platelet aggregation [10]. *Taraxacum officinale* is suggested as food source because of the high content of minerals, fiber, vitamins, and essential fatty acids [11]. Anti-inflammatory effects of *Taraxacum officinale* and its effect on downregulation of No, PGE_(2)_, and proinflammatory cytokines are due to phenolic compound that reduced expressions of iNos and cox-2 via inactivation of the MAP kinase signal pathway [12]; therefore according to similar studies on herbs such as Nettle [13] maybe *Taraxacum officinale* causes inhibition of production of PGE from arachidonic acid and so inhibits of production thromboxane. As in Figure 2, rate of WBC in groups 1 (treated with dose of 50 mg/kg), 2 (treated with dose of 100 mg /kg), and 3 (treated with dose of 200 mg/kg) than the control group has increased, but these differences only in group 3 are significant, and as the Figure 3 indicated that rate of lymphocyte in these experimental groups 1, 2, 3 than the control group has increased significantly. The reason of this increasing is due to anti-virus effects [14], antiinflammatory effects [15] and antibacterial effects [16] of this plant. According to Singh study and colleagues in 2008, phenolic compounds in dandelion such as chicoric acid inhibit the penetration of viruses in cells. Nitric oxide is important for immune regulation, and defense) this molecule can be inhibited by cadmium. An aqueous extract of *Taraxacum officinale* has been shown to overcome this inhibitory effect of cadmium and work in a dose-dependent manner to restore nitric oxide production by mouse peritoneal macrophages and caused enhancing cell-mediated, humoral, and nonspecific immunity [10, 16]. This effect is thought to be primarily a result of the extract's ability to induce the secretion of tumor necrosis factor-alpha [17, 18]. The crude extract of dandelion leaf decreased the growth of MCF-7AZ breast cancer cells and blocked the invasion of LNCap prostate cancer cells, also dandelion root extract blocked invasion of MCF-7AZ breast cancer cells [18]. Finally, the results can say that hydroalcoholic extract of dandelion can be effective on blood factors. More research is recommended.
